# The Effects of an *In Vitro* Oocyte Maturation System and Chlorogenic Acid Supplementation during Embryo Culture on the Development of Porcine Cloned Embryos Derived from Native Vietnamese Ban Pigs

**DOI:** 10.1155/2023/5702970

**Published:** 2023-04-17

**Authors:** Thanh Van Nguyen, Lanh Thi Kim Do, Ngoc-Anh Thi Nguyen, Kazuhiro Kikuchi, Tamas Somfai, Takeshige Otoi

**Affiliations:** ^1^Department of Animal Theriogenology and Surgery, Faculty of Veterinary Medicine, Vietnam National University of Agriculture, Hanoi, Vietnam; ^2^The Graduate School of Veterinary Science, Yamaguchi University, Yamaguchi, Japan; ^3^Institute of Agrobiological Sciences (NIAS), National Agriculture and Food Research Organization (NARO), Tsukuba, Japan; ^4^Faculty of Bioscience and Bioindustry, Tokushima University, Tokushima, Japan

## Abstract

The aim of this study was to improve the production efficiency of Vietnamese native Ban pig embryos using somatic cell nuclear transfer (SCNT). Fibroblast cells from Ban pigs were injected into the enucleated cytoplasts of crossbred gilts, and the reconstructed embryos were subsequently cultured. In the first experiment, cytoplasts were isolated from oocytes matured in either a defined porcine oocyte medium (POM) or in TCM199 medium supplemented with porcine follicular fluid. Both media were supplemented with gonadotropic hormones, either for the first 22 h of *in vitro* maturation (IVM) or for the entire 44 h of IVM. In the second experiment, the reconstructed SCNT embryos were cultured with or without 50 *μ*M chlorogenic acid (CGA). Furthermore, this study examined parthenogenetic embryos. The IVM medium and duration of hormone treatment did not affect embryo development. CGA supplementation to the culture medium significantly increased blastocyst formation rates in parthenogenetic embryos but not in SCNT embryos. However, CGA supplementation significantly reduced the apoptotic index in blastocysts regardless of embryo source. In conclusion, the IVM method did not affect SCNT embryo production, while CGA supplementation during embryo culture improved the quality of SCNT embryos in indigenous pig breeds.

## 1. Introduction

Somatic cell nuclear transfer (SCNT) is a potential tool for the preservation of porcine genetic resources since it allows the production of offspring from cryopreserved somatic cells maintained in gene banks[1]. Furthermore, SCNT in conjunction with genome editing has been regarded as an effective method for both agricultural and biomedical research [2]. In terms of genome sequences, anatomy, and physiology, pigs are quite similar to humans, making them an excellent research model [3], a potential source of organs for xenotransplantation [4], and potential bioreactors for producing hyperimmune sera [5]. Smaller minipig models are easier to handle, have lower operating costs than typical pig models, and have higher ethical acceptability for widespread usage in agriculture and research [6, 7]. The Vietnamese Ban pig is a miniature breed with a lower porcine endogenous retrovirus (PERV) copy number than Western breeds [8]. However, since the outbreak of African swine fever in Vietnam worsened the status of local pig breeds, the development of SCNT techniques, which allow for progeny from somatic cells stored in gene banks, is urgently needed.

Throughout the last decade, significant efforts have been made to improve cloning efficiency in mammals [9–11], including optimising IVM and *in vitro* culture (IVC) medium. Nevertheless, SCNT of indigenous pig breeds has rarely been performed in Vietnam and is still characterised by relatively low embryo production rates [12]. The aim of this study was to improve the efficiency of SCNT embryo production from native Vietnamese Ban pigs. The IVM and IVC systems have an impact on the efficiency of SCNT embryo production [13]. As for the IVM of porcine oocytes, both the basic medium and its supplementation with gonadotropins influence subsequent embryonic development [14]. In a recent study, porcine oocyte medium (POM) was reported to be better than the cheaper NCSU-37 for the IVM of porcine oocytes utilized for SCNT [12]. Nevertheless, in the study, the feasibility of TCM-199 (which is cheaper than POM but more complex than NCSU-37) was not investigated. In addition, the optimum duration for hormone exposure has been evaluated in traditional IVM and *in vitro* fertilisation (IVF) systems [15, 16] but not for SCNT procedures. As a result, in the first part of the present study, we examined the effectiveness of embryo production by SCNT following IVM of porcine oocytes in TCM199 and POM coupled with two gonadotropin treatment protocols.

Previously, we demonstrated that supplementation of the medium with chlorogenic acid (CGA), a phenolic antioxidant, during IVM could improve developmental competence following IVF or electrostimulation of porcine oocytes [17–19]. However, it is unknown whether CGA supplementation in an IVC medium is a potent approach for improving SCNT embryo development. Therefore, in the second part of the present study, we evaluated the effects of CGA supplementation in an IVC medium on the development and quality of porcine SCNT embryos.

## 2. Materials and Methods

As no live animals were used in this study, ethical approval was not required at any of the participating institutions.

### 2.1. Preparation of Donor Cells

Fibroblast cells isolated from ear tissues of Vietnamese Ban pigs were cultured in DMEM medium supplemented with 10% (v/v) foetal bovine serum (FBS, Sigma-Aldrich, MO, USA) and 50 *μ*g/mL gentamicin at 37°C in a humidified atmosphere containing 5% CO_2_. Confluent fibroblast monolayers were washed twice with DMEM containing gentamicin (washing medium) and then incubated in 0.25% trypsin-EDTA (Sigma-Aldrich) for 3 min at 37°C. Subsequently, the cells were washed via centrifugation at 500 ×*g* for 5 min and maintained in a washing medium until SCNT manipulation. Fibroblast cells from passages 2–5 of the culture were used as donor cells.

### 2.2. Oocyte Collection and IVM

Porcine ovaries were obtained from prepubertal crossbred gilts (Landrace × Yorkshire breeds) at a local slaughterhouse and transported to the laboratory within 1 h in physiological saline (0.9% (w/v) NaCl) at 30°C. The ovaries were washed three times with modified phosphate-buffered saline (m-PBS; Sigma-Aldrich, MO, USA) supplemented with 100 IU/mL penicillin G potassium (Sigma-Aldrich) and 0.1 mg/mL streptomycin sulfate (Sigma-Aldrich). Cumulus-oocyte complexes (COCs) were collected by dissecting follicles 3–6 mm in diameter in Medium 199 with Hank's salts (Gibco, CA, USA) supplemented with 10% FBS (Gibco; Invitrogen Corp., Carlsbad, CA, USA), 20 mM HEPES (Sigma-Aldrich) and antibiotics (100 U/mL penicillin G potassium (Sigma-Aldrich) and 0.1 mg/mL streptomycin sulfate (Sigma-Aldrich). After dissection, only COCs with uniformly dark-pigmented ooplasm and intact cumulus cell masses were collected. Approximately 50 COCs were cultured in 500 *μ*L of maturation medium with mineral oil (Sigma-Aldrich) in 4-well culture dishes (SPL, Gyeonggi, Korea). All incubations were performed in a humidified incubator containing 5% CO_2,_ 5% O_2,_ and 90% N_2_ in the air at 38.5°C.

According to the experimental design, two types of basic media were used in this study. Half of the collected COCs were cultured in TCM maturation medium, consisting of 25 mM HEPES tissue culture medium 199 with Earle's salts (TCM 199; Invitrogen, USA), supplemented with 10% (v/v) porcine follicular fluid (pFF), 0.6 mM cysteine (Sigma-Aldrich), 50 *μ*M sodium pyruvate (Sigma-Aldrich), 2 mg/mL D-sorbitol (Wako, Japan), 1 *μ*g/mL 17*β*-estradiol (Sigma-Aldrich), and 50 *μ*g/mL gentamicin (Sigma-Aldrich). Another half of the remaining oocytes were matured in a defined porcine oocyte medium (POM; [20]) supplemented with 3 mg/mL polyvinyl alcohol (PVA), 10 ng/mL epidermal growth factor (eGF, Sigma-Aldrich), and 1.0 mM/L dibutyryl cyclic adenosine monophosphate sodium salt (dbcAMP, Sigma-Aldrich).

For both types of IVM media, the two gonadotropin treatment protocols were compared according to the experimental design. The medium was supplemented with 10 IU/mL equine chorionic gonadotropin (Kyoritsu Seiyaku, Japan) and 10 IU/mL human chorionic gonadotropin (Kyoritsu Seiyaku) during the entire 44 h of IVM or during the first 22 h of IVM, which was followed by an additional 22 h of culture without gonadotropin supplementation.

### 2.3. SCNT and Parthenogenetic Stimulation of IVM Oocytes

SCNT was conducted as previously described by Onishi et al. [21] with minor modifications. After IVM, pig COCs were denuded by exposure to 150 IU of hyaluronidase (Sigma-Aldrich) and mechanical pipetting. Oocytes with the first polar body were collected and then incubated for 10 min in 3 *μ*g/mL of Hoechst 33342 (Sigma-Aldrich) diluted in manipulation medium containing 5 *µ*g/mL cytochalasin B in porcine zygote medium 3 (PZM3; [22]) medium. The removal of metaphase II chromosomes and the first polar body from oocytes was then gently performed by aspiration with a flat-tip micropipette (8–12 *μ*m internal diameter) driven by a piezo-actuated unit (Prime Tech, Ibaraki, Japan). Enucleation success was confirmed by examination under ultraviolet light. Each donor cell was subsequently injected into the cytoplasm of an enucleated oocyte, and the reconstructed embryos were electro-activated in an activation solution containing 280 mM D-mannitol, 0.05 mM CaCl_2_, 0.1 mM MgSO_4_, and 0.01% (w/v) BSA with a single 100 *μ*s activating pulse of 1.5 kV/cm in the LF500G1 electro-chamber (BEX, Tokyo, Japan) connected to an LF101 cell fusion unit (BEX, Tokyo, Japan).

Parthenogenetic stimulation of IVM oocytes was performed as described by Iwamoto et al. [23]. Briefly, polar body-extruded oocytes were transferred to an activation solution and stimulated with a direct current pulse, as mentioned above. Following electroactivation, the reconstructed embryos and activated oocytes were transferred to a PZM3 medium supplemented with cytochalasin B (5 *µ*g/mL) for 2 h, after which the oocytes were cultured described as follows.

### 2.4. Embryo Culture and Assessment of Blastocyst Quality

The oocytes were cultured in 50-*µ*L PZM3 medium in 6-well culture dishes (IFP9670, Japan) overlaid with mineral oil for 7 days. On day 5 (Day 0 = the day of stimulation), the culture drops were supplemented with 10% (v/v) prewarmed FBS (Sigma-Aldrich). Cleavage and blastocyst formation were evaluated under a stereomicroscope on Day 2 and Day 7, respectively. To evaluate the total cell number and frequency of apoptotic cells, blastocysts were fixed on Day 7 and subjected to a combined technique for simultaneous nuclear staining and terminal deoxynucleotidyl transferase nick-end labelling (TUNEL), as described previously [24]. Briefly, the blastocysts were fixed overnight at 4°C in 3.7% (w/v) paraformaldehyde diluted in PBS. After fixation, the cells were permeabilised in 0.1% (v/v) Triton-X100 dissolved in PBS for 40 min and then incubated overnight at 4°C in PBS containing 10 mg/mL BSA (blocking solution). The cells were then incubated in fluorescein-conjugated 2-deoxyuridine 5-triphosphate and TUNEL reagent (Roche Diagnostics Corp., Basel, Switzerland) for 1 h at 38.5°C. The blastocysts were then placed on glass slides in glycerol and counterstained with 1 *µ*g/mL bisbenzimide (Hoechst 33342; Sigma-Aldrich) before being overlaid with coverslips and sealed with clear nail polish. TUNEL and Hoechst staining were visualized using an epifluorescence microscope (BX53; Olympus, Tokyo, Japan) at excitation wavelengths of 550 nm and 350 nm, respectively. Apoptotic nuclei exhibit condensed and fragmented morphology [25], as labelled by green TUNEL staining. The apoptotic index was calculated by dividing the number of cells with apoptotic nuclei by the total number of cells.

### 2.5. Experimental Design

#### 2.5.1. Experiment 1

To evaluate the effects of maturation medium and hormone exposure duration on the developmental competence of Ban pig SCNT embryos, COCs were matured either in TCM-199 or POM medium as described above (TCM and POM groups, respectively). In both groups, COCs matured in the presence of gonadotropic hormones during the entire 44 h of IVM or the first 22 h of IVM. After IVM, oocytes from each group were used for SCNT, and subsequent embryo development was compared among the groups, as described above.

#### 2.5.2. Experiment 2

To evaluate the effects of CGA supplementation during IVC, both SCNT and parthenogenetic embryos produced from oocytes matured in POM medium were cultured in PZM3 medium supplemented with 0 (control group) or 50 *μ*M CGA (Sigma-Aldrich). In Experiment 1, POM medium with hormone treatment for the first 22 h of IVM was found to be most suitable for the development of embryos. The CGA concentration in this experiment was determined according to our previous reports [17–19]. The culture medium was PZM3 without hypotaurine, which may act as a potential antioxidant because the positive effects of the antioxidant CGA may be veiled by other potential antioxidant reagents in the culture medium. Embryo development and blastocyst quality were assessed, as described above.

### 2.6. Statistical Analysis

Each experiment was replicated at least six times. The percentage data for oocyte maturation (polar body extrusion), cleavage, blastocyst formation, and apoptotic nuclei were subjected to arcsine transformation before the analysis. Two-way ANOVA was used to examine the relationship (interaction) of two main factors (IVM medium and hormone exposure duration in Experiment 1 and embryo source and IVC medium in Experiment 2) and their relationship (interaction) on the transformed data and the total cell numbers of blastocysts. When any significant differences were revealed, detailed analyses of the effects among the treatment groups were carried out using the protected Fisher's least significant difference test using the StatView software package (Abacus Concepts, Berkeley, CA, USA). Differences with a probability value of *P* ≤ 0.05 were considered statistically significant.

## 3. Results

### 3.1. Effects of Maturation Medium and Hormone Exposure Duration

There was no statistically significant difference in oocyte maturation rates (the proportion of oocytes with the first polar body) among the groups (Table 1). The rates of cleavage and blastocyte formation were likewise comparable among the four experimental groups. However, the blastocyst formation rate of SCNT embryos derived from oocytes matured in POM medium with hormone exposure for 22 h tended to be higher than that of embryos derived from oocytes matured in POM with hormone exposure for 44 h (20.1% vs. 12.4%, *P*=0.068).

### 3.2. Effects of CGA Supplementation during IVC

When evaluating the percentages of embryo development and quality, we detected embryo source × IVC medium interactions in the cleavage and apoptotic indices (*P* < 0.05) (Table 2). The presence of CGA during IVC improved the blastocyst formation rate of parthenogenetic embryos (40.3%) compared with that of embryos cultured without CGA (23.7%, *P* < 0.05). However, there was no statistically significant difference in blastocyst formation rates between SCNT embryos cultured with and without CGA. Moreover, the presence of CGA during IVC successfully restored the apoptotic indices in both parthenogenetic blastocysts (8.5% vs. 14.2%, *P* < 0.01) and SCNT blastocysts (12.3% vs. 19.8%, *P* < 0.01), even though total cell numbers in the blastocysts were not significantly different between the groups.

## 4. Discussion

In the present study, we isolated Ban pig somatic cells from ear tissues as donor cell for SCNT and assessed the effects of oocyte maturation and embryo culture medium on the development and quality of SCNT embryos. Our results suggest that POM and TCM-199 are equally feasible as base media during IVM, as highlighted by similar rates of oocyte maturation and embryo development when the two media were compared using the same hormone treatment protocol. Hormone treatment during IVM, including the duration of exposure, promotes cumulus cell expansion and stimulates oocyte maturation, influencing the fertilisation rate and embryo development [15, 26]. In addition, under *in vitro* conditions, the addition of hormones during maturation helps to synchronise the development of the nucleus and cytoplasm in oocytes [27]. Funahashi and Day [15] reported in a conventional IVF system that hormone exposure during IVM for 20, 30, and 40 h significantly increased oocyte maturation rates compared to those at 0 or 2 h of hormone exposure, in which 20 h of hormone exposure improved the proportion of oocytes with male and female pronuclei (normal fertilisation). Schoevers et al. [16] also suggested that the addition of FSH during the first 20 h of IVM culture was the most beneficial for porcine oocyte maturation and embryonic development after IVF. In the present study, we first evaluated the effect of the duration of hormone treatment on the development of porcine SCNT embryos. However, unlike in earlier reports applying IVF, there was no significant difference in the rates of oocyte maturation, cleavage, and blastocyst formation among the groups, regardless of the maturation medium and hormone treatment duration. Porcine IVM/IVF systems are often affected by high frequencies of fertilisation abnormalities, such as failure of male pronuclear formation and/or polyspermy [28]. Thus, the beneficial effects of optimised IVM systems are attributed to the ability of oocytes to undergo normal fertilisation events [15, 16]. In contrast, the use of SCNT to generate embryos (which does not involve sperm penetration) eliminates the effects of fertilisation events. Therefore, the difference in the effects of hormone exposure on developmental competence of embryos between IVF reports and SCNT results may be attributed to the use of SCNT. Nevertheless, the blastocyst formation rate of SCNT embryos derived from oocytes matured in POM medium with hormone exposure during the first half (22 h) of IVM tended to be higher than that attained for the entire 44 h of IVM. Therefore, IVM in POM combined with exposure to gonadotropic hormones during the first 22 h of the IVM period was applied in Experiment 2.

The developmental competence of porcine IVP embryos is significantly lower than that of embryos produced *in vivo* as well as those of other species derived *in vitro* [29]. Many issues remain with porcine IVP systems, and despite these efforts, IVC conditions have not been completely optimised in this species. One of the remaining problems is the sensitivity of porcine embryos to oxidative stress during IVC [28]. Chlorogenic acid is a phenolic antioxidant that improves the development of IVF-derived and parthenogenetic porcine embryos when supplemented during IVM [17–19]. As we did not detect any significant influence of the IVM medium and hormone supplementation on the developmental competency of SCNT embryos, we decided to investigate the effects of CGA supplementation to the media during embryo culture on the development and quality of SCNT and parthenogenetic embryos. Our results showed that CGA supplementation during IVC significantly improved the blastocyst formation rate of parthenogenetic embryos but not that of SCNT embryos. However, the apoptotic index in blastocysts was diminished by the presence of CGA, regardless of the source of the embryos. Apoptosis, a type of programmed cell death, occurs naturally during preimplantation embryonic development [30]. The apoptotic index in preimplantation embryos is considered one of the most important parameters for evaluating embryo quality [25, 31]. During IVC, porcine embryos react to high levels of oxidative stress with an increased apoptotic index [32]. A previous study reported that porcine SCNT embryos have a higher apoptotic incidence than IVF embryos [33]. Moreover, Hao et al. [34] demonstrated that the low developmental competence of SCNT-derived embryos was associated with a higher number of apoptotic cells, which increased with IVC time. For this reason, in previous studies, transfers of pig SCNT embryos to recipients were performed during early embryonic development between the one-cell and eight-cell stages [13, 35]. Our results demonstrate that supplementation of embryo culture medium with the antioxidant CGA is a possible approach to reduce the apoptotic index of SCNT blastocysts, indicating the potential of CGA to improve the quality of SCNT-derived blastocysts.

In conclusion, the media and hormone treatment protocol used during IVM did not affect embryo production by SCNT. However, our results demonstrated the positive effects of CGA supplementation during embryo culture on the quality of porcine SCNT and parthenogenetic embryos. The results indicate the possibility of improving the developmental potential of SCNT embryos in indigenous pig breeds by preventing the activation of the apoptotic pathway, especially during the preimplantation embryo stage.

## Figures and Tables

**Table 1:** Effects of maturation medium and hormone exposure duration on oocyte maturation and developmental competence of Ban Pig somatic cell nuclear transfer (SCNT) embryos^*∗*^.

IVM mediums	Duration of hormone supplementation (h)	Number (%) of oocyte		Number (%) of SCNT embryos		
		Cultured	With a polar body (%)	Fused	Cleaved (%)	Developed to the blastocyst stage (%)
POM	22	329	274 (83.6 ± 3.8)	149	87 (59.1 ± 5.1)	30 (20.1 ± 3.3)
POM	44	307	248 (81.4 ± 3.7)	143	83 (56.9 ± 4.3)	18 (12.4 ± 2.4)
TCM	22	320	242 (76.0 ± 3.8)	149	81 (53.4 ± 3.4)	22 (14.3 ± 2.9)
TCM	44	318	240 (76.0 ± 4.5)	135	79 (59.4 ± 3.9)	22 (16.4 ± 2.8)

^*∗*^All experiments were repeated seven times. Percentage data are expressed as mean ± SEM.

**Table 2:** Effects of CGA supplementation during *in vitro* culture (IVC) on the development and quality of Ban pig somatic cell nuclear transfer (SCNT) embryos^*∗*^.

Embryo sources	IVC medium	Number (%) of embryos			Number of blastocysts examined	Total cell number	Apoptotic index (%)^*∗∗*^
		Cultured	Cleaved	Developed to blastocysts			
Parthenogenesis	PZM3	119	109 (91.6 ± 4.0)	29 (23.7 ± 4.8)^a^	28	40.4 ± 3.9	14.2 ± 1.3^A^
	PZM3 + CGA	120	115 (95.8 ± 1.5)	47 (40.3 ± 7.8)^b^	30	36.7 ± 5.3	8.5 ± 1.2^B^

SCNT	PZM3	135	113 (83.9 ± 2.2)	35 (26.1 ± 5.5)^a,b^	23	38.3 ± 2.9	19.8 ± 2.3^A^
	PZM3 + CGA	138	111 (80.4 ± 2.3)	33 (23.8 ± 2.9)^a^	21	39.5 ± 5.6	12.3 ± 1.2^B^

^*∗*^All experiments were repeated six times. Percentage data and cell numbers are expressed as mean ± SEM. ^*∗∗*^Apoptotic index was calculated by dividing the number of cells containing apoptotic nuclei by the total number of cells. ^a,b^Values with different superscripts in the same column are significantly different (*P* < 0.05). ^A,B^A embryo source × IVC medium interaction was detected in the cleavage and apoptotic indices (*P* < 0.05). Values with different superscripts in the same embryo source are significantly different (*P* < 0.05).

## Data Availability

The data used to support the findings of this study are included within the article.
